# Draft genomes of three strains of *Pseudomonas aeruginosa* from clinical, recreational, and ocean surface water samples

**DOI:** 10.1128/mra.01468-25

**Published:** 2026-02-13

**Authors:** Abraham Goodness Ogofure, Ezekiel Green

**Affiliations:** 1 Department of Biotechnology and Food Technology, University of Johannesburg, Doornfontein Campus 61799, Johannesburg, South Africa; 2 Microbial Pathogenicity and Molecular Epidemiology Research Group (MPMERG), Department of Biotechnology and Food Technology, University of Johannesburg, Doornfontein Campus 61799, Johannesburg, South Africa; Rochester Institute of Technology, Rochester, New York, USA

**Keywords:** clinical isolate, swimming pool, otitis externa, bacterial adaptability, antimicrobial resistance, ocean water, recreational water

## Abstract

We describe draft genomes of three *Pseudomonas aeruginosa* strains from aquatic and clinical settings in South Africa. Assemblies show high completeness, consistent GC content, distinct sequence types, and varied virulence and resistance genes. These genomes extend representation of environmental and human-associated *P. aeruginosa* and enable comparative analyses across local ecological and exposure routes.

## ANNOUNCEMENT

*Pseudomonas aeruginosa* is a gram-negative, rod-shaped bacterium, an adaptable opportunist that bridges environmental and clinical settings (1, 2). It colonizes diverse environments with extensive study in clinical context (1, 3–5); comparatively little information exists for recreational waters (2). We present draft genomes of three *P. aeruginosa* strains from distinct ecological niches in Sodwana Bay, sequenced to expand exposure-route representation, after identifying clinically relevant resistance and virulence traits in environmental strains. Together with prior marine and swimmer isolates (1, 2), these genomes enable regional comparison of environmental and human-associated strains.

Isolates were recovered on nutrient and *Pseudomonas* cetrimide agar (Oxoid, UK) and incubated at 37°C for 24–48 h. Colonies with morphology consistent with *P. aeruginosa* were sub-cultured and initially identified using standard biochemical tests (non-fermentative, oxidase positive). Molecular confirmation used genomic DNA as a template for 16S rRNA gene amplification with universal primers and *P. aeruginosa* species-specific primers, followed by multilocus sequence typing (MLST) of the seven housekeeping genes (*acsA*, *aroE*, *guaA*, *mutL*, *nuoD*, *ppsA*, and *trpE*) according to the *P. aeruginosa* MLST scheme (1). For whole-genome sequencing, isolates were grown overnight in Luria–Bertani broth at 37°C with shaking (180 rpm). Genomic DNA was extracted using the NucleoSpin Microbial DNA kit (Macherey-Nagel, Germany) according to the manufacturer’s instructions, and DNA purity and concentration were assessed with a NanoDrop ND-2000 spectrophotometer (Thermo Fisher Scientific, USA) (Table 1). Sequencing libraries were prepared for paired-end sequencing and run on an Illumina MiSeq platform (2× 300 bp), generating 1.2–2.5 million paired-end reads per isolate. Raw reads were assessed with FastQC (v.0.69) and quality-filtered and adapter-trimmed using Trimmomatic (v.0.39), retaining bases with Phred scores ≥20 (1, 6). Filtered reads were assembled *de novo* with Unicycler (v.0.4.1.1) using default parameters, and assembly statistics were calculated with QUAST (v.4.6.3). Genome annotation was performed using the NCBI Prokaryotic Genome Annotation Pipeline and the RAST server. Assembly quality was further evaluated with CheckM to estimate completeness and contamination. Antimicrobial resistance genes and virulence factors were screened using curated databases (e.g., CARD/NDARO for resistance and VFDB/PATRIC for virulence) with minimum thresholds of ≥90% sequence identity and ≥80% coverage (1, 6–8). Average nucleotide identity (ANI) was calculated using FastANI implemented via the Bioconda distribution, comparing assembled genomes against the *P. aeruginosa* PAO1 reference strain (GCF_000006765.1).

**TABLE 1:** Summary of genome assembly and quality metrics for three *P. aeruginosa* isolates from distinct environments in Sodwana Bay

Isolate_ID	Source (Date)	Genome size (Mb)	G + C content (%)	Contigs (n)	Completeness (%)	Genome coverage	Scaffold N50 (L50)	Contamination (%)	GenBank predicted genes (n)
BDPW	Swimming pool (2020)	6.4	66.5	443	98.53	10×	25.7 kb (77)	0.24	6,189
BIS	Ocean surface water (2020)	6.7	66.0	288	97.9	10×	37.2 kb (55)	0.20	6,323
BIS1	Clinical isolate (otitis externa) (2020)	6.7	66.0	442	98.46	10×	25.6 kb (81)	0.41	6,434

All three genomes exhibited ANI values exceeding 98% relative to the *P. aeruginosa* PAO1 reference strain, confirming species-level assignment. All isolates shared a high GC content, estimated completeness, and low contamination, as assessed by CheckM. BIS displayed the highest contiguity, whereas BDPW and BIS1 were more fragmented. MLST assigned BDPW to ST971 and BIS1 to ST244; BIS was non-typeable due to a missing aroE allele. BIS1 encoded the largest predicted gene set and contained CRISPR arrays. Each genome carried antimicrobial resistance determinants and virulence factors typical of *P. aeruginosa*, including efflux pumps, β-lactamase genes, and type III secretion effectors (*exoS*/*exoU*). Despite similar sequencing depth (~10×), they share a conserved genomic backbone with variation in sequence type and accessory gene content. These findings support the ecological plasticity of *P. aeruginosa* at the human–environment interface and highlight the value of continued One Health genomic surveillance of recreational waters and associated clinical cases.

These draft assemblies are suitable for comparative genomics, AMR surveillance, and phylogenetic analysis but may have limitations for detailed structural variant detection and complete plasmid characterization because complete assemblies would require long-read sequencing. No additional read error-correction or post-assembly polishing was performed.

## Data Availability

The whole-genome shotgun projects for the three strains have been deposited in GenBank under BioProject PRJNA695104, with genome assembly accession numbers GCA_016820275.1 (BDPW), GCA_016820195.1 (BIS), and GCA_016820175.1 (BIS1), corresponding to BioSample accessions SAMN17601188, SAMN17601191, and SAMN17601192, respectively. Raw sequencing reads are available in the Sequence Read Archive under accession numbers SRR35978001 (BDPW), SRR35978000 (BIS), and SRR35977999 (BIS1). All data are publicly accessible through the NCBI database at https://www.ncbi.nlm.nih.gov.

## References

[1] Serepa-Dlamini MH, Kondiah K, Maumela P, Ogofure AG, Green E. 2025. Genomic insights into five selected multidrug-resistant Pseudomonas aeruginosa isolated from Sodwana Bay, South Africa. Front Microbiol 16:1578578. doi:10.3389/fmicb.2025.1578578 40673149 PMC12263573

[2] Maclean K, Njamo FOJP, Serepa-Dlamini MH, Kondiah K, Green E. 2022. Antimicrobial susceptibility profiles among Pseudomonas aeruginosa isolated from professional SCUBA divers with otitis externa, swimming pools and the ocean at a diving operation in South Africa. Pathogens 11:91. doi:10.3390/pathogens11010091 35056039 PMC8777857

[3] Silby MW, Winstanley C, Godfrey SAC, Levy SB, Jackson RW. 2011. Pseudomonas genomes: diverse and adaptable. FEMS Microbiol Rev 35:652–680. doi:10.1111/j.1574-6976.2011.00269.x 21361996

[4] Stover CK, Pham XQ, Erwin AL, Mizoguchi SD, Warrener P, Hickey MJ, Brinkman FS, Hufnagle WO, Kowalik DJ, Lagrou M, et al.. 2000. Complete genome sequence of Pseudomonas aeruginosa PAO1, an opportunistic pathogen. Nature 406:959–964. doi:10.1038/35023079 10984043

[5] Moradali MF, Ghods S, Rehm BHA. 2017. Pseudomonas aeruginosa lifestyle: a paradigm for adaptation, survival, and persistence. Front Cell Infect Microbiol 7:39. doi:10.3389/fcimb.2017.00039 28261568 PMC5310132

[6] Bogaerts B, Delcourt T, Soetaert K, Boarbi S, Ceyssens P-J, Winand R, Van Braekel J, De Keersmaecker SCJ, Roosens NHC, Marchal K, Mathys V, Vanneste K. 2021. A bioinformatics whole-genome sequencing workflow for clinical Mycobacterium tuberculosis complex isolate analysis, validated using a reference collection extensively characterized with conventional methods and in silico approaches. J Clin Microbiol 59:e00202-21. doi:10.1128/JCM.00202-21 33789960 PMC8316078

[7] Brown VI, Lowbury EJL. 1965. Use of an improved cetrimide agar medium and other culture methods for Pseudomonas aeruginosa. J Clin Pathol 18:752–756. doi:10.1136/jcp.18.6.752 4954265 PMC473125

[8] Purushothaman S, Meola M, Roloff T, Rooney AM, Egli A. 2024. Evaluation of DNA extraction kits for long-read shotgun metagenomics using Oxford Nanopore sequencing for rapid taxonomic and antimicrobial resistance detection. Sci Rep 14:29531. doi:10.1038/s41598-024-80660-3 39604411 PMC11603047

